# Copy‐number analysis of Y‐linked loci in young men with non‐obstructive azoospermia: Implications for the rarity of early onset mosaic loss of chromosome Y

**DOI:** 10.1002/rmb2.12321

**Published:** 2020-03-02

**Authors:** Erina Suzuki, Yoshitomo Kobori, Momori Katsumi, Kikumi Ushijima, Toru Uchiyama, Hiroshi Okada, Mami Miyado, Maki Fukami

**Affiliations:** ^1^ Department of Molecular Endocrinology National Research Institute for Child Health and Development Tokyo Japan; ^2^ Department of Urology Dokkyo Medical University Saitama Medical Center Koshigaya Japan; ^3^ Department of NCCHD Child Health and Development Graduate School Tokyo Medical and Dental University Tokyo Japan; ^4^ Department of Human Genetics National Research Institute for Child Health and Development Tokyo Japan

**Keywords:** azoospermia, chromosome deletion, karyotype, sex chromosome, Y‐linked gene

## Abstract

**Purpose:**

Mosaic loss of chromosome Y (mLOY) is a common feature in elderly men. If mLOY can also occur in young men, it may lead to spermatogenic failure due to loss of spermatogenic genes. Indeed, previous studies detected the 45,X/46,XY karyotype in a few young men with spermatogenic failure. The present study aimed to clarify the frequency of cryptic mLOY in reproductive‐aged men with spermatogenic failure.

**Methods:**

We studied 198 men at ages 24‐55 years who presented with etiology‐unknown non‐obstructive azoospermia. Prior this study, these patients underwent G‐banding analysis for 20 leukocytes and were found to have 46,XY karyotype. We analyzed copy numbers of chromosome Y in blood cells by using semi‐quantitative multiplex PCR for *AMELY*/*AMELX*, array‐based comparative genomic hybridization (CGH) for the *AMELY* locus, and droplet digital PCR for *SRY*, *USP9Y*, and *UTY*.

**Results:**

Multiplex PCR showed borderline low *AMELY/AMELX* ratios in three patients. However, for the three patients, CGH excluded deletion of the *AMELY* locus, and droplet digital PCR suggested preserved copy numbers of all tested loci.

**Conclusion:**

This study highlights the rarity of leukocyte mLOY in reproductive‐aged men with spermatogenic failure. In addition, our data imply that standard karyotyping is sufficient to screen early onset mLOY.

## 1 INTRODUCTION

Human chromosome Y is often lost in the somatic cells of elderly men.^1^ Recent studies have shown that the frequency of recognizable mosaic loss of chromosome Y (mLOY), that is, LOY involving ≥10% of peripheral blood cells, continuously increases with age in men over 40 years of age.^1^, ^2^, ^3^ Aging‐related mLOY has been associated with shorter life expectancy and increased risks of cancer and other disorders.^1^, ^4^, ^5^ The frequency of mLOY in men under 40 years of age remains unknown, but is predicted to be extremely low.^4^, ^5^

Human chromosome Y encodes several spermatogenic genes.^6^ Hemizygous interstitial deletions of chromosome Y, particularly those in the azoospermia factor (AZF) region, are known as the risk factor of non‐obstructive azoospermia.^7^ Thus, mLOY, if it occurs in testis of young men before or during reproductive ages, may cause spermatogenic failure. In 2016, Shin et al^8^ performed standard karyotyping for blood samples from 1,354 reproductive‐aged men with non‐obstructive azoospermia and detected a mosaic 45,X/46,XY karyotype in eight. Similarly, Yatsenko et al^9^ identified the same karyotype in one of 629 samples from men with severe spermatogenic failure. These results provided the first indication that early onset mLOY in leukocytes is present in a small percentage of young men with spermatogenic failure. Given that standard karyotyping subjects only about 20 leukocytes,^8^ this method may miss some cases with mLOY. Hence, the frequency of mLOY among patients may have been underestimated in the previous studies. The present study aimed to clarify the frequency of cryptic mLOY in young men with spermatogenic failure. To this end, we screened 198 samples using a semi‐quantitative multiplex PCR method, whose sensitivity has been confirmed in previous studies.^10^, ^11^, ^12^

## 2 MATERIALS AND METHODS

### 2.1 Patients

The study group consisted of 198 Japanese patients with non‐obstructive azoospermia at ages 24‐55 years (median 34 years). The patients were numbered in order of their age. These patients visited our clinic because of infertility and were diagnosed with non‐obstructive azoospermia of unknown etiology. Prior to the present study, all patients underwent conventional G‐banding analysis for 20 peripheral leukocytes and were found to have a normal 46,XY karyotype. In addition, the patients were subjected to copy‐number analysis of the AZF regions,^7^, ^13^ which showed the lack of AZF deletion in 132 of 198 patients. The common gr/gr, AZFb, AZFc, and AZFb+c deletions were detected in 49, 1, 15, and 1 patient(s), respectively. The frequencies of these AZF deletions were comparable to previous data in Japan.^7^

### 2.2 Screening of mLOY by semi‐quantitative multiplex PCR

We performed semi‐quantitative multiplex PCR for all samples to screen mLOY. Genomic DNA samples were obtained from peripheral blood of each patient. We utilized a previously established method with slight modifications.^10^ In brief, copy numbers of chromosome Y relative to chromosome X were assessed by comparing the area under the curve of PCR products for *AMELY* at Yp11.2 to that for *AMELX* at Xp22.2. The PCR products were analyzed using the 3130 Genetic Analyzer with LIZ500 (Applied Biosystems). The area under the curve was calculated using the GeneMapper 3.7 software (Applied Biosystems). Samples which showed *AMELY*/*AMELX* ratios of ≤0.89 in two independent assays were subjected to the second analysis by droplet digital PCR. This cutoff value, 0.89, was determined based on the previous report^11^ and our own data on 42 fertile men aged between 31 and 39 years (range, 0.89‐1.04; mean, 0.97).

### 2.3 Detection of copy‐number alterations involving ***AMELY***

Low *AMELY*/*AMELX* ratios are indicative of mLOY; however, they can also result from interstitial deletions involving the *AMELY* locus. To exclude such Y‐linked interstitial deletions*,* we performed array‐based comparative genomic hybridization (CGH) for samples with a low *AMELY*/*AMELX* ratio. We used a human catalog array (4 × 180 k format; Agilent Technologies, Palo Alto, CA), which contains three probes for the *AMELY* gene. Copy number of the *AMELY* locus was assessed using the Genomic Workbench (version 7.0.4.0, Agilent technologies) with the default settings of the aberration detection algorithm.

### 2.4 Detection of mLOY by droplet digital PCR

To examine the copy number of chromosome Y in the samples with possible mLOY, we performed droplet digital PCR.^14^ Genomic DNA samples were analyzed using the QX200 system (Bio‐Rad Laboratories). We examined copy numbers of three loci, that is*, SRY* (Bio‐Rad Laboratories, Assay ID: dHsaCP2500472), *USP9Y* (Assay ID: dHsaCP2506328), and *UTY* (Assay ID: dHsaCNS782024066). *RPP30* at 10q23.31 (Assay ID: dHsaCP2500350) was utilized as the internal reference. For each locus, two independent assays were performed.

## 3 RESULTS

### 3.1 Screening of mLOY by semi‐quantitative multiplex PCR

**Figure 1:**
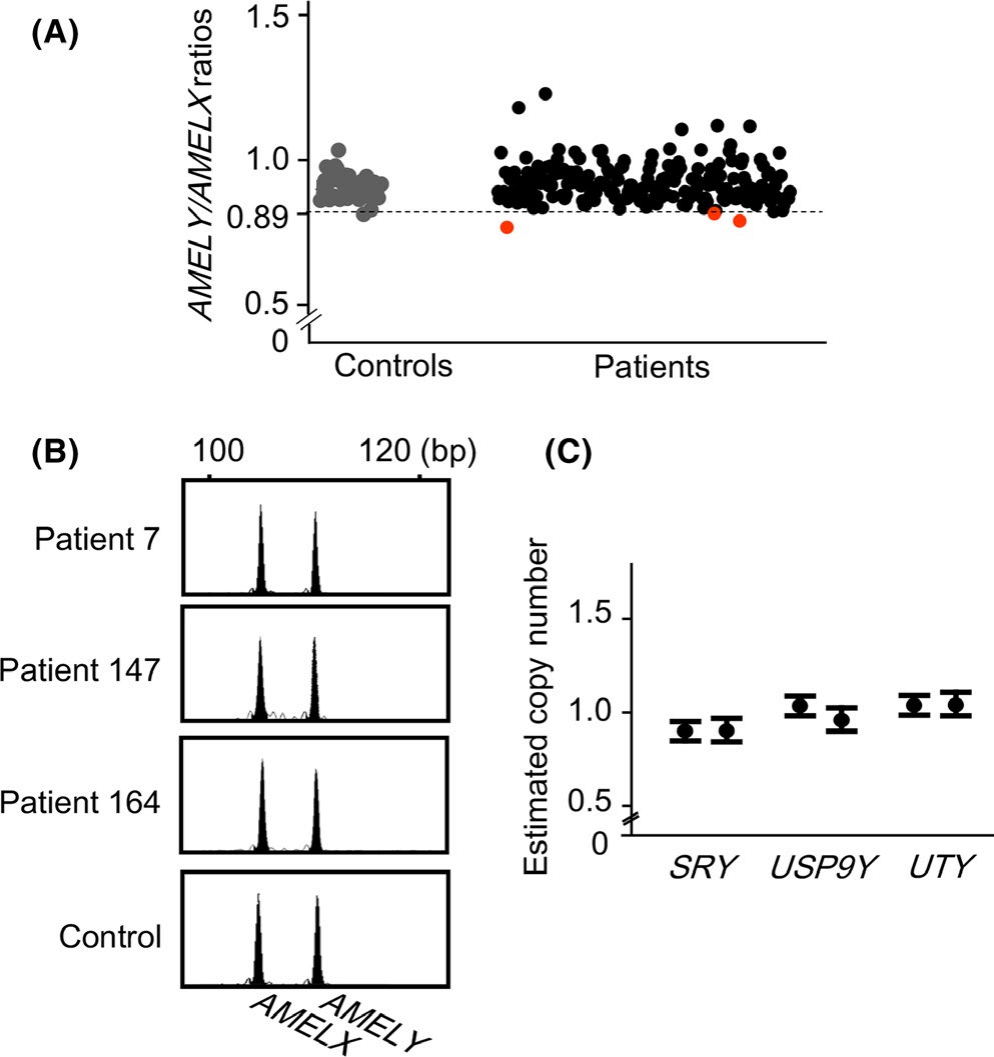
Representative results of molecular analyses. A, Results of semi‐quantitative multiplex PCR for each participant. The gray, red, and black dots indicate the *AMELY/AMELX* ratios in control fertile males (n = 42), three patients with low ratios (patients 7, 147, and 164), and the remaining patients (n = 195), respectively. Broken line indicates the cutoff value (0.89). B, Raw data of semi‐quantitative multiplex PCR for patients 7, 147, and 164. The sizes and amounts of PCR products of the *AMELY* and *AMELX* loci are shown. We calculated the area under the curve for the two loci. As a control, the results of a 32‐year‐aged fertile man are shown. C, Results of droplet digital PCR for patient 164. The dots and the paired bars indicate the estimated copy numbers of *SRY*, *USP9Y,* and *UTY*, and the 95% confidence intervals, respectively. The results of two independent assays are shown

Among the 198 patients examined, three (patients 7, 147, and 164) exhibited low *AMELY/AMELX* ratios. The three patients had no AZF microdeletion. Average *AMELY/AMELX* ratios of patients 7, 147, and 164 were 0.85, 0.89, and 0.87, respectively (Figure 1A,B), whereas the ratios of the remaining 195 samples ranged between 0.90 and 1.26.

### 3.2 Detection of copy‐number alterations involving ***AMELY***

Array‐based CGH for patients 7, 147, and 164 detected no copy‐number alterations at the *AMELY* locus (Figure S1). Thus, the low *AMELY/AMELX* ratios in these patients were not ascribable to an interstitial deletion of chromosome Y.

### 3.3 Detection of mLOY by droplet digital PCR

Droplet digital PCR for patients 7, 147, and 164 showed apparently normal copy numbers for the three tested loci (Figure 1C). Thus, these patients were unlikely to have mLOY.

## 4 DISCUSSION

None of the 198 men with non‐obstructive azoospermia and normal karyotype had recognizable mLOY. Whereas semi‐quantitative multiplex PCR showed low *AMELY*/*AMELX* ratios in three patients, droplet digital PCR for these individuals suggested normal copy numbers of three loci on chromosome Y. The semi‐quantitative multiplex PCR method have been used in several previous studies^10^, ^11^, ^12^ showing that its detection limit of mLOY is as low as 4.6%.^10^ Thus, it is unlikely that this method failed to detect leukocyte mLOY in our subjects. In this regard, the false‐positive results of patients 7, 147, and 164 were not ascribable to interstitial deletion of the *AMELY* locus, because array‐CGH demonstrated a normal copy number for this locus. The low *AMELY*/*AMELX* ratios of these patients may reflect the relatively low specificity of this screening method. Actually, the ratios in patients 7, 147, and 164 were only slightly lower than the cutoff value.

We cannot completely exclude the possibility that some of our 198 subjects carried mLOY exclusively in the testis. Indeed, previous studies detected mLOY not only in blood cells, but also in buccal samples.^15^, ^16^, ^17^ Considering that strong expression of the sex‐determining gene *SRY* was observed in the fetal testis but not in the postnatal testis,^18^ postnatal mLOY of young men may impair spermatogenesis but likely permits normal male‐type sexual development. However, the probability of testis‐specific occurrence of mLOY is low, because mLOY is predicted to occur predominantly in high‐turnover cells such as peripheral leukocytes.^1^, ^2^

The results of this study, in conjunction with the previous reports by Shin et al^8^ and Yatsenko et al^9^ in which standard karyotyping detected mLOY in 0.60% and 0.15% of azo/oligozoospermia patients, respectively, indicate that leukocyte mLOY accounts for an extremely small fraction of young men with spermatogenic failure. Since no data are currently available for the frequency of leukocyte mLOY in healthy men under 40 years of age, it remains unknown whether early onset mLOY is more common in patients with spermatogenic failure than in the general population. Furthermore, there are no data whether mLOY in young men is associated with early death and the risk of various disorders, as is the case for elderly people.^1^, ^4^, ^5^ In this context, Thompson et al^3^ raised questions about the pathogenicity of mLOY in blood cells. It is possible that leukocyte mLOY itself has no deleterious effects on men's health, but reflects genomic instability in other tissues. Large‐scale studies are needed to clarify the clinical significance of leukocyte mLOY in young men.

In summary, the results of this study demonstrated the rarity of mLOY in reproductive‐aged men with spermatogenic failure. Thus, early onset mLOY is unlikely to play a major role in the development of spermatogenic failure. In addition, our data imply that standard karyotyping is sufficient to screen early onset mLOY.

## CONFLICT OF INTEREST

The authors declared that they have no competing interests.

## HUMAN RIGHTS STATEMENTS AND INFORMED CONSENT

This study was approved by the Institutional Review Board Committee at the National Center for Child Health and Development. All procedures followed were performed in accordance with the Helsinki Declaration of 1964 and its later amendments. Written informed consent was obtained from all participants.

## ANIMAL STUDIES

No animals were used in this study.

## Supporting information

FigS1
