# Supplementary figures and images for: Copy‐number analysis of Y‐linked loci in young men with non‐obstructive azoospermia: Implications for the rarity of early onset mosaic loss of chromosome Y
Source: Reprod Med Biol. 2020 Mar 2;19(2):178–81. doi: 10.1002/rmb2.12321 (PMC7138941; doi:10.1002/rmb2.12321)

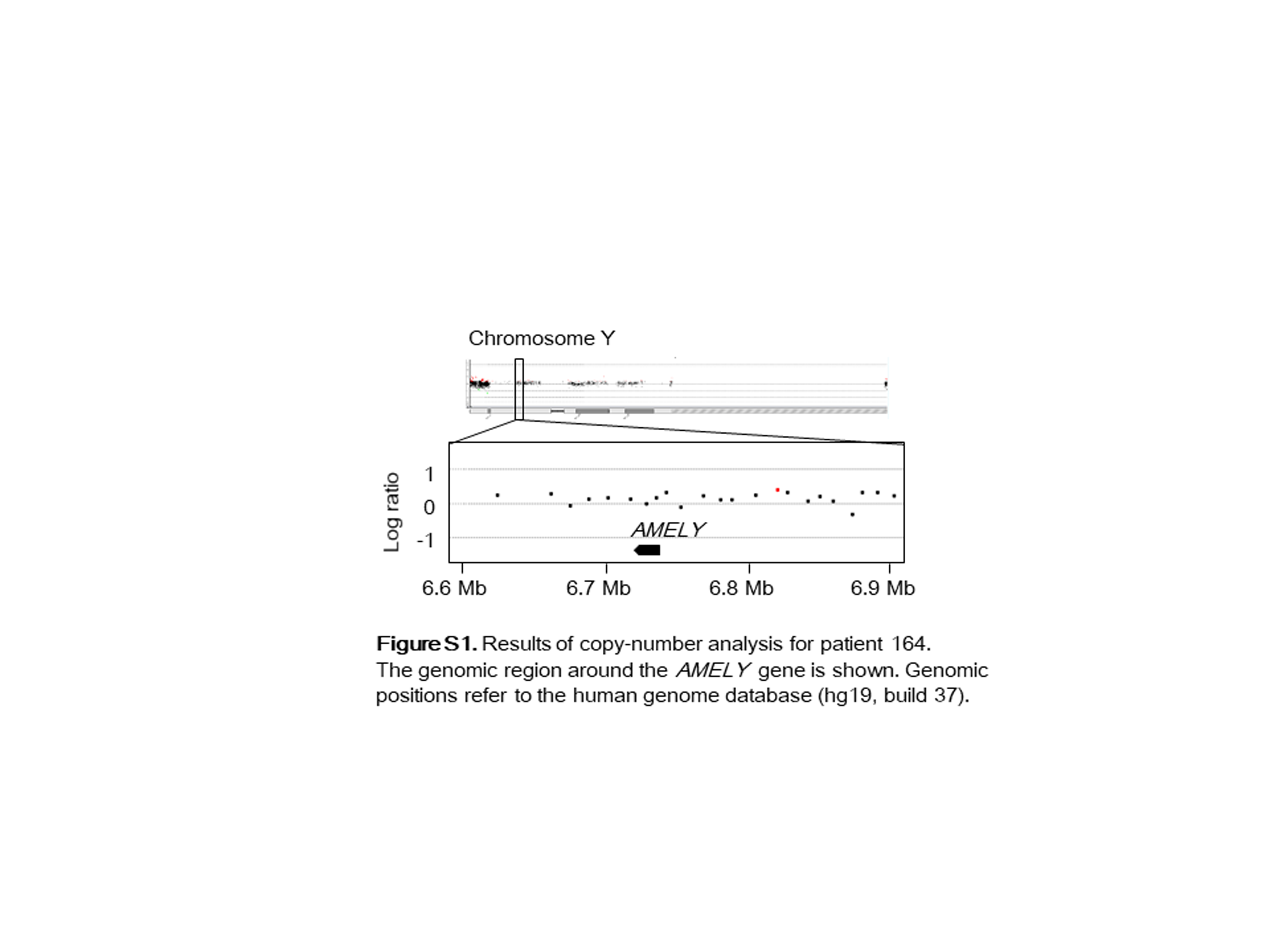

Supplement: Supplementary file 1 — FigS1 [file RMB2-19-178-s001.tif]
